# Genomics and transcriptomics reveal β-carotene synthesis mechanism in *Dunaliella salina*

**DOI:** 10.3389/fmicb.2024.1389224

**Published:** 2024-05-17

**Authors:** Duo Chen, Zhenhui Li, Jiaxian Shi, Huamiao Suen, Xuehai Zheng, Cifeng Zhang, Youqiang Chen, Ting Xue

**Affiliations:** The Public Service Platform for Industrialization Development Technology of Marine Biological Medicine and Products of the State Oceanic Administration, Fujian Key Laboratory of Special Marine Bioresource Sustainable Utilization, Center of Engineering Technology Research for Microalga Germplasm Improvement of Fujian, Southern Institute of Oceanography, Fujian Normal University, Fuzhou, China

**Keywords:** *Dunaliella salina*, genome, transcriptome, β-carotene, synthesis mechanism

## Abstract

*Dunaliella salina* is by far the most salt-tolerant organism and contains many active substances, including β-carotene, glycerol, proteins, and vitamins, using in the production of dried biomass or cell extracts for the biofuels, pharmaceutical formulations, food additives, and fine chemicals, especially β-carotene. We report a high-quality genome sequence of *D. Salina* FACHB435, which has a 472 Mb genome size, with a contig N50 of 458 Kb. A total of 30,752 protein-coding genes were predicted. The annotation results evaluated by BUSCO was shown that completeness was 91.0% and replication was 53.1%. The fragments were 6.3% and the deletions were 2.6%. Phylogenomic and comparative genomic analyses revealed that *A. thaliana* diverged from Volvocales about 448 million years ago, then *Volvocales C. eustigma*, *D. salina*, and other species diverged about 250 million years ago. High light could promote the accumulation of β-carotene in *D. salina* at a 13 d stage of culture. The enrichment of DEGs in KEGG, it notes that the predicted up-regulated genes of carotenoid metabolic pathway include *DsCrtB, DsPDS, DsZ-ISO, DsZDS, DsCRTISO, DsLUT5, DsCrtL-B*, and *DsCCD8*, while the predicted down-regulated genes include *DsCrtF*, and *DsLUT1*. The four genes that were both up-regulated and down-regulated were *DsZEP, DsCrtR-b, DsCruA/P* and *DsCrtZ 4*. The research results can provide scientific basis for the industrialization practice of *D. salina*.

## Introduction

**Figure 1:**
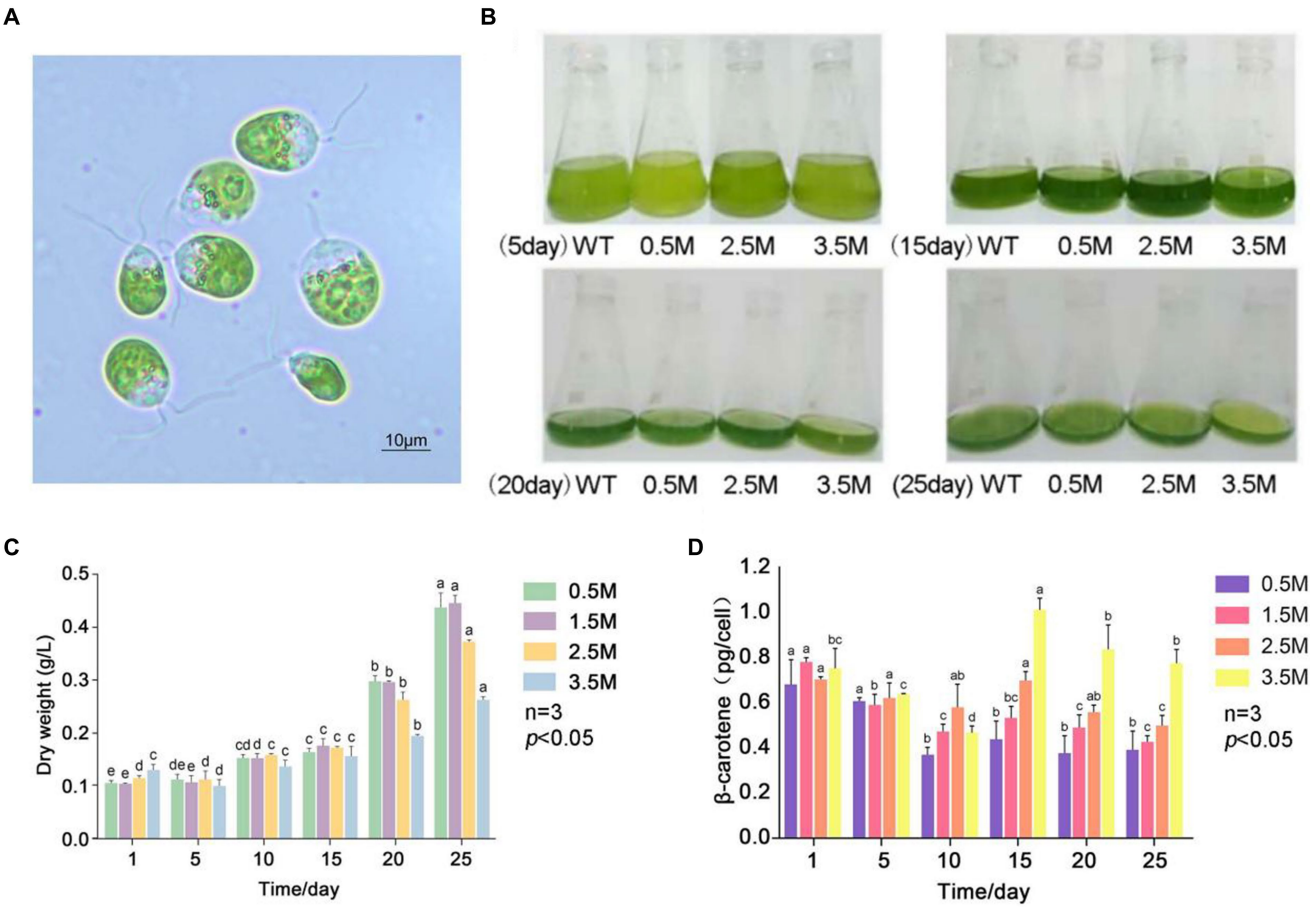
β-carotene content under salinity stress in *D. salina* FACHB-435. **(A)** Light microscopy images of *D. salina* FACHB435. **(B)** Variations in *D. salina* FACHB-435 across various developmental phases with differing salt levels. **(C)** The dry mass of *D. salina* FACHB-435 during distinct growth periods at various salinity concentrations. **(D)** Levels of β-carotene in *D. salina* FACHB-435 through different stages of growth influenced by changing salt concentrations.

*Dunaliella salina* is a single-cell organism in the family Chlorophyaceae. It lacks a true cell wall but is wrapped in a glycoprotein and has two flagella that can swim freely in liquid environments (Figure 1A) (Leliaert et al., 2012). It can grow normally at concentrations of NaCl ranging from 0.05 M to saturated and regulates the osmotic pressure inside and outside the cell by regulating glycerol metabolism. *D. salina* is by far the most salt-tolerant organism and contains many active substances, including glycerol, beta-carotene, proteins, and vitamins. It is used in the production of dried biomass or cell extracts for the biofuels, pharmaceutical formulations, food additives, and fine chemicals (Brown, 1991). Most animals cannot synthesize carotenoids automatically and must consume plant foods to meet the demand for carotenoids. Carotenoids are used in the animal body to improve the immune system, inhibit and prevent cancer, delay aging, improve liver damage, and act as nutritional antioxidants that promote communication between cellular junctions, which can effectively inhibit the occurrence of some chronic diseases (VanEenwyk et al., 1991; Berman et al., 2015). Among them, beta-carotene is a vital precursor for the synthesis of vitamin A in humans and animals, which is also an antioxidant (Harrison, 2005). Beta-carotene inhibits and prevents tumors, controls cholesterol, and reduces the incidence of cardiovascular disease (Lee et al., 1999). In the last decade, some genes related to carotenoid biosynthesis have been studied in organisms such as *Haematococcus pluvialis*, *Chlorella zofingiensis*, and *Chlamydomonas reinhardtii* (Peter et al., 1992; Hirschberg et al., 1997; Schmidt-Dannert, 2000). As a direct or indirect natural bait for fish, shrimp, and shellfish larvae or adults, microalgae are of great interest in the development of new aquafeed resources because they are rich in protein, fat, polysaccharides, vitamins, antioxidant substances, pigments, trace elements, and other nutrients (Nishida et al., 2023). The biochemical and genetic basis of carotenoid biosynthesis has been well studied in terrestrial plants and various microorganisms, but most of the studies on carotenoid synthesis in algae have focused on microalgae, especially *Chlamydomonas* (Huang et al., 2017).

Light intensity, nutrient deficiencies, salinity, hormones, and other environmental factors can influence the production of carotenoids in microalgae. Specific types of carotenoids can be produced in response to different environmental stimuli by genes involved in carotenoid biosynthesis, and certain genes in the metabolic pathway can act synergistically to regulate their production. In recent years, the production of bio-organic compounds such as β-carotene has been established (Raja et al., 2007). In the 1980s, large-scale salt algae farms were established in the United States, Australia, and other countries, resulting in significant production from algal breeding, harvesting, and extraction of cultured β-carotene. *D. salina* has been shown to accumulate three valuable products: carotene, glycerol, and protein. Under specific conditions, salt algae can contain up to 14% β-carotene (Ye et al., 2008), while under inappropriate conditions, the β-carotene content is only 0.3% or less (Johnson and Schroeder, 1996). Studies have shown that the highest carotenoid production in *D. salina* CCAP 19/18 occurs under high light intensity and nitrogen limitation conditions (Park et al., 2013). Researchers investigated the effects of nitrogen concentration, salinity, and light intensity on the production of lutein in *D. salina* and found that nitrogen limitation and high salt had a strong synergistic effect. β-Carotene, a tetraterpene synthesized from isopentenyl pyrophosphate (IPP) and dimethylallyl pyrophosphate (DMAPP) (Schwender, 1996), serves as a vital precursor to vitamin A, offering benefits like antioxidation, immune system support, reduction of cardiovascular disease risk, and cancer prevention. Its extensive applications span the pharmaceutical, healthcare, and food sectors (Burton Graham et al., 2014). *D. salina*, capable of thriving under environmental stress, significantly boosts its carotenoid levels in such conditions, presenting substantial nutritional and commercial interest. Investigating the molecular basis for β-carotene accumulation in this halophilic microalgae, coupled with advances in molecular genetic techniques, holds promise for enhancing β-carotene production. Efforts to identify and clone candidate genes from *D. salina* have been limited in the past by the lack of a reference genome with high-quality gene annotations and by the absence of suitable transcriptomic data for co-expression analysis. Recently, there has been a genome study of *D. Salina*, in which the assembled genome strain of *D. Salina* is ACC, whereas the strain we studied is FACHB435. The predicted genome size of FACHB435 is more than 100 M larger than that of the previous ACC strain. Thus, we assembled a high-quality genome of FACHB435 to conduct further transcriptome differential gene mining and gene function overexpression studies.

In this study, we constructed a chromosomal-level genome of *D. salina* FACHB435 by integrating PacBio, Illumina, and Hi-C sequencing strategies. The generated datasets, based on the assembled genomic and transcriptomic data, genome-wide annotation, and genome-wide replication analysis, were used to perform evolutionary analysis of the species genomes. We also performed transcriptomic analysis for *D. salina* under light stress to identify differentially expressed genes. The results of this study can serve as a valuable resource for future *D. salina* genomic studies, promoting downstream analysis and research of *D. salina* related genes. Investigating the molecular basis behind the accumulation of β-carotene in *D. salina* through molecular biology approaches, along with enhancing β-carotene yields via genetic breeding techniques, offers considerable nutritional and commercial benefits.

## Materials and methods

### Sample materials, cultures and growth conditions

The strain *D.salina* FACHB-435 was acquired from the Freshwater Algae Culture Collection at the Institute of Hydrobiology (FACHB) in Wuhan, Hubei. *D. salina* strain was cultured in liquid DM medium with a trace element solution diluted to 10% with Milli-Q water, and the pH value was adjusted to 7.5 after medium preparation. The cultures were incubated at a temperature of 25 ± 2°C with a photoperiod of 12/12 h light/dark cycle, and the culture bottles were shaken at least 6 times a day to ensure proper mixing. To isolate a sterile *D. salina* cell, techniques such as capillary separation, plate marking, and antibiotic sterilization were employed. The identification of *D. salina* FACHB-435, through both morphological and molecular biology methods, verified its classification within the *Dunaliella* genus (Figure 1A). The algal liquid cultured to the later stage of logarithmic growth was centrifuged and inoculated into the newly prepared medium with the same amount. The normal light (NL) 2,500 Lux as control groups and the high light (HL) 10,000 Lux as treatment groups were set, and three replicates were set for each experimental group. The inoculated algal liquid was placed on the light culture rack at the culture temperature of (25 ± 1) °C, the light and dark period L/D was 12 h/12 h, and the bottle was shaken 6 times a day. After inoculation, the growth state of algal cells was observed. At 0, 5, 13 and 20 days of culture, algal fluid was taken for dry weight, β-carotene content detection and RNA sequencing, respectively.

### Determination of β-carotene content by HPLC

The determination of β-carotene in *D. salina* by high-performance liquid chromatography according to the following process: a 10 mL aliquot of algal suspension was centrifuged at 5,000 rpm for 5 min, the supernatant was discarded, and the algal biomass was transferred to a 2 mL Eppendorf tube. The sample was flash-frozen in liquid nitrogen for a few seconds before being stored in a − 80°C freezer for several hours and then freeze-dried using a lyophilizer. The freeze-dried algal powder was stored in a − 80°C freezer. To the algal powder, 2 mL of acetone was added, and the mixture was ground using a grinder at a frequency of 50 Hz for 3 min, until the algal powder turned white. After allowing the mixture to settle for 20 min, the supernatant was collected, centrifuged, and filtered through a 0.22 μm organic membrane into an HPLC sample vial. Since *D. salina* cells have a high lipid content that can interfere with β-carotene extraction, causing incomplete extraction or emulsification, a saponification reaction was performed prior to pigment extraction. The lipid extract was dissolved in 1 mL of toluene, and 100 μL of 0.1 g/mL KOH-methanol saponifying agent was added. The mixture was allowed to stand at room temperature for 1 h. The supernatant was then collected, centrifuged, and filtered through a 0.22 μm organic membrane into an HPLC sample vial. The sample was stored at −80°C until further analysis.

The samples were analyzed using an Agilent 7890B gas chromatograph equipped with a capillary column (HP88, 100 m, 0.25 mm, 0.2 μm film thickness) and a hydrogen/air flame ionization detector (FID). Helium served as the carrier gas at a constant flow rate of 20 mL/min. The injector temperature was maintained at 250°C, and a 1 μL sample volume was injected with a 10: 1 split ratio. The temperature program started at 140°C with a 5-min hold, then increased to 240°C at a rate of 4°C/min, followed by a 12.5-min hold. The mass spectrometer (MS) operated in electron impact ionization mode with an electron energy of 70 eV. The other parameters included: full scan mode with an m/z range of 50–550; ion source temperature at 230°C; and quadrupole temperature at 150°C. The National Institute of Standards and Technology (NIST) library database was used to identify the components. The standard curve was prepared using C8-22 fatty acid methyl esters and quantified by the internal standard method. Each experiment was independently replicated three times.

### Genomic DNA extraction

Genomic DNA was isolated from 100 mL of fresh culture. The cell suspension was centrifuged at 8000 rpm for 5 min. The cell pellet was then suspended in approximately 1 mL of medium and pipetted into a 2-mL tube and centrifuged again at 8000 rpm for 5 min. Briefly, 800 μL of 2% mercaptoethanol solution was pipetted into each sample, followed by the addition of 800 μL of 10% w/v CTAB (in 0.7 M NaCl solution) and incubation at 56°C for 10 min. After extraction with an equal volume of phenol: chloroform: isoamyl alcohol (25:24:1), the mixture was centrifuged at 12,000 rpm for 10 min twice. The supernatant was dissolved in 0.4 mL of 100 μg/mL RNase and incubated at 37°C for 30 min. An equal volume of chloroform: isoamyl alcohol (24:1) was then centrifuged at 12,000 rpm for 10 min. Genomic DNA was precipitated by adding 2.5 volumes of 100% ethanol and collected by spinning at 12,000 rpm and 4°C for 10 min. After the supernatant was discarded, the resulting genomic DNA pellet was stored in 5.0 mL of 70% cold ethanol at 4°C overnight to allow the impurity to dissolve. Finally, DNA was eluted in 100 μL of 10 mM Tris HCl by centrifugation at 12,000 rpm for 1 min. The purity and concentration of DNA were analyzed using a NanoDrop 2000 Spectrophotometer (Thermo Scientific, United States). More than 5 μg of sheared and concentrated DNA was applied to size-selection by the BluePippin system. Approximately 20-kb SMRTbellTM libraries were prepared according to the released protocol from PacBio company. A total of 6.7 Gb subreads were sequenced on the PacBio sequel system, i.e., 106 × coverage of the estimated genome size. After removing the low-quality (containing 10 or more Ns and low-quality bases with quality scores ≤7) and redundant reads.

### Genome sequencing and *de novo* assembly

In this study, Jellyfish software (Marçais and Kingsford, 2011) was used to analyze the distribution of k-mer. FACHB-435 genome was assembled based on the third-generation PacBio data, and the FACHB-435 initial genome was corrected using the second-generation Illumina data. Based on the third-generation sequencing data, we first compared two sets of third-generation assembly software:Canu (Koren et al., 2017) and Mecat (Xiao et al., 2017).

### Evaluate the genome assembly

BUSCO (Viridiplantae_odb10), EST, and Illumina alignment were used to evaluate the completeness and quality of the assembled genome (Camacho et al., 2009; Langdon, 2015; Simão et al., 2015). BUSCO software is used for the accuracy and integrity of genome assembly, gene sets, and transcripts. BUSCO software. The OrthoDB database constructs single-copy gene sets for several large evolutionary branches. The gene set is compared with the assembled genome, and the accuracy and completeness are assessed according to the ratio and completeness of the comparison. Using the complete transcript, mapping to the sequence of the spliced sample is used to assess the integrity of the gene coding region.

### Transcriptome annotation and analysis

The repetitive sequence prediction of *D. salina* first uses RepeatModeler to obtain a denovo repeat sequence library, which can automatically execute the two denovo search software RECON and RepeatScout, after obtaining the consistent TE, import the result into RepeatMasker to identify and cluster the repeat sequences. Repeat sequences are classified using a process for identifying them (Flynn et al., 2020). In order to obtain high-quality genes, this project used *de novo* prediction (Augustus, SNAP, etc.) and homologous prediction software (GeneWise) combined with transcriptome data to predict gene structure.

First, RNAseq samples were selected and imported into Trinity *de novo* assembly and Trinity genome-guided assembly (Grabherr et al., 2011). Then RSEM was used to calculate transcript abundance (filter out FPKM< 1 and iso-percentage < 3%; Filtered transcripts are imported into PASA software to build more comprehensive transcripts). PASA is able to detect new transcripts using high sensitivity based on reference assembly, combined with the ability to assemble from scratch, and then train the identified transcripts as parameters. By comparison with the Uniprot plant protein database, transcripts with protein coverage greater than 95% were retained as candidate transcripts. Then, the gene prediction software SNAP, GENEMARK and AUGUSTUS based on *ab initio* theory were used to extract and retrain the gene model with AED score of 0 (Holt and Yandell, 2011), and the final gene prediction result was obtained by integrating MAKER software (Stanke et al., 2006). Through gene structural annotation, a total of 30,752 protein-coding genes were predicted. The predicted gene structure was evaluated by BUSCO.

### Evolution and phylogeny of the genome

By employing OrthoFinder (version 2.3.12), orthologous genes were found between D.salina and 12 species, including Cyanidioschyzon merolae, Porphyriopsis chorda, Gracilariopsis chorda, *Porphyra umbilicalis*, *Isochrysis galbana*, *Emiliania huxleyi*, Phaeodactylum tricomutum, Chlamydomonas rheinhardtii, *Thalassiosira pseudonana*, Chlamydomonas eustigma, *Gonium pectorale*, Volvox carteri. The maximum likelihood tree was constructed with rhodophyta as an outgroup. Homologous base pairs between species were identified using jcvi’s compara tool (Tang et al., 2015). Then, according to the synonymous_calculation module in the tool bio-pipeline, synonymy replacement rate (Ks) distribution of homologous base pairs obtained in the previous step was analyzed, and the whole genome replication events were inferred (Zhang et al., 2006; Rodamilans et al., 2014).

### Total RNA isolation, library construction, and sequencing

21 samples were used for RNA sequencing: Dsa-0d1, Dsa-0d2, Dsa-0d3, Dsa-NL5d1, Dsa-NL5d2, Dsa-NL5d3, Dsa-NL13d1, Dsa-NL13d2, Dsa-NL13d3, Dsa-NL20d1, Dsa-NL20d2, Dsa-NL20d3 (control: samples cultured under the normal light 2,500 Lux), and Dsa-HL5d1, Dsa-HL5d2, Dsa-HL5d3, Dsa-HL13d1, Dsa-HL13d2, Dsa-HL13d3, Dsa-HL20d1, Dsa-HL20d2 and Dsa-HL20d3 (treatment: samples cultured under the high light 10,000 Lux). Total RNA was extracted by the instruction manual of RNA extraction reagent kit Trans Zol Up Plus RNA Kit (Beijing TransGen Biotech Co., Ltd). The transcriptome library were constructed and sequenced using the Illumina Hiseq4000 platform.

### Expression quantification and differential expression analysis

The expression level of the gene is represented by the FPKM value, which represents the number of sequenced fragments included in every thousand sequencing bp per million sequencing bases. The Cufflinks v2.2.1 software was used to calculate the expression level of the genes (Roberts et al., 2011). After calculating the expression levels of all genes, we identified and statistically analyzed the differentially expressed genes (DGEs) between the control group and high-light stressed *D. salina* at two time points (0dVs5dNL, 5dNLVs13dNL, 13dNLVs20dNL, 0dVs5d-HL, 5d-HL Vs13d-HL, 13d-HL Vs20d-HL), searched for common DEGs, and performed clustering analysis on all DEGs. The standard for defining DEGs is: *p*-Value<0.05, Fold change>2 (Nikolayeva and Robinson, 2014). DEGs can be divided into two categories based on their relative expression levels: Up-regulated Genes and Down-regulated Genes.

### qRT-PCR validation

Using the RNA from the control and experimental groups on the 5th and 13th days of culture as templates, reverse transcription was performed according to the instructions of the TransScript One-Step gDNA Removal and cDNA Synthesis SuperMix kit(Beijing TransGen Biotech Co., Ltd.) The obtained cDNA was used as a template, and fluorescence quantification detection was performed using the ABI Step One fluorescent quantitative PCR instrument, according to the instructions of the Quanshijin TransStart®TOP Green qPCR SuperMix kit. We chose three genes, ***Ds**LCYB*, ***Ds**PDS* and ***Ds**ZDS*, to validate.

## Results

### Effect of salinity and light intensity on growth and β-carotene accumulation of *Dunaliella salina*

We investigated the growth and β-carotene accumulation patterns of *D. salina* under four salinity gradients: 0.5 M, 1.5 M, 2.5 M, and 3.5 M (Figure 1B). Figure 1C shows the dry weight of algae at different salinity gradients, with the dry weight in each group increasing as culture time progressed. The dry weight of the low salinity 0.5 M group and the control group were similar, while the high salinity experimental group consistently had a lower dry weight than the control group. Under high salinity conditions, cell numbers increased slowly and cell density remained lower than in the control group. Figure 1D presents the β-carotene content of *D. salina* FACHB-435 at different salinities. The β-carotene content per cell initially decreased and then gradually increased during normal culture, stabilizing as culture time increased. Upon resuming culture, the β-carotene content per cell increased with salinity. On day 15, the high salt stress group’s β-carotene content per cell was significantly higher than that of the control group, with an increase of 72.15% (*p* < 0.01). The figure also displays the biomass of *D. salina* under different light intensity conditions, revealing that the biomass increases with light intensity. It can be observed that, on the 10th day of incubation, the β-carotene accumulation in individual algal cells under high light intensity was significantly higher than that in the control group. As incubation time increased, β-carotene accumulation in single cells decreased, with higher light intensities correlating to lower β-carotene content (Figure 1D).

### *Dunaliella salina* FACHB435 genome size

Based on the 17-K-mer results, it showed that the haploid genome size of of *D. salina*, we FACHB-435 was 386.49 Mb. Genome homozygosity (100%- genome heterozygosity) was 99.713%, homozygosity > 99%, indicating high homozygosity of genome (Supplementary Table S1). Only a single peak indicates low genome complexity, and there is no obvious peak behind the main peak, which further indicates that the repeat sequence is low, indicating good sequencing results (Supplementary Figure S1).

### Genome assembly and annotation for of *Dunaliella salina*

In this study, genomes were assembled with three-generation data and corrected with second-generation data. After two analysis of three-generation assembly software, it is obvious that the assembly of MECAT software (Genome Size: 472 Mb N50:458 kb) is better than the assembly results of CANU software (Genome Size: 511 Mb N50:185 kb) (Table 1). Our assessment of genomic assembly complete showed that the overall complete BUSCOs was 91.0%, where complete and single-copy BUSCOs 42.2%, complete and duplicated BUSCOs 48.8%. The proportion of complete and duplicated BUSCOs is relatively high. Fragmented BUSCOs was 6.3%, Missing BUSCOs 2.7% (Supplementary Table S2).

**Table 1:** Genome assembly results statistics.

Iterms	CANU	MECAT
Number of seq	5,115	1,623
Total size	510,616,574	471,590,659
N90	45,834	138,267
N80	77,514	208,153
N70	111,706	283,429
N60	146,838	363,446
N50	185,470	458,221
Average length	99,827	290,567
Total number(>500 bp)	5,115	1,623
Total number(>2 kb)	5,084	1,623

The annotation results were evaluated by BUSCO, as shown Supplementary Table S3. After the whole genome annotation is completed, 303 lineal homologous gene sets in eukaryota_odb9 are evaluated. The results showed that completeness was 74.2%, complete and single-copy BUSCOs was 27.7%, and complete and duplicated BUSCOs 46.5%, which was slightly higher. The fragments were 3.3% and the missing BUSCOs 22.5%. The integrity of gene annotation is high, which can be used for metabolic studies related to *D. salina* (Supplementary Table S3).

The repetitive sequence prediction of *D. salina* first uses RepeatModeler to obtain a *de novo* repeat sequence library, which can automatically execute the two *de novo* search software RECON and RepeatScout, after obtaining the consistent TE, import the result into RepeatMasker to identify and cluster the repeat sequences. The process of identifying repeat sequences was used to classify them, and the resulting repeat sequence classification was shown in Supplementary Table S4.

RNAseq samples were selected and imported into Trinity *de novo* assembly and Trinity genome-guided assembly. Then RSEM was used to calculate transcript abundance (filter out FPKM<1 and iso-percentage < 3%). Filtered transcripts are imported into PASA software to build more comprehensive transcripts. PASA is able to detect new transcripts using high sensitivity based on reference assembly, combined with the ability to assemble from scratch, and then train the identified transcripts as parameters. By comparison with the Uniprot plant protein database, transcripts with protein coverage greater than 95% were retained as candidate transcripts. Then, the gene prediction software SNAP, GENEMARK and AUGUSTUS based on *ab initio* theory were used to extract and retrain the gene model with AED score of 0, and the final gene prediction result was obtained by integrating MAKER software. Through gene structural annotation, a total of 30,752 protein-coding genes were predicted. The predicted gene structure was evaluated by BUSCO, and the results were shown in Supplementary Table S5. By BUSCO analysis (Supplementary Table S5), the total completeness was 75.9%, with 44.9% gene replication accounting for a slightly higher proportion. Fragmented is only 4% and Missing 20.1%. On the whole, if the deletion of conserved genes within the species is not taken into account, the quality of the genome assembled by us is medium.

According to the evaluation results, the replication integrity of *D.salina* takes up 44.9% of the total, which is analyzed by RepeatMasker software, as shown in Supplementary Table S5. The Total repeat fraction is 43.75% of the genome, accounting for about half of the genome, among which the Class-I of TEs accounts for a relatively high proportion, accounting for 30.25% of the genome. The specific proportion distribution of TEs in repeated sequences is shown in Supplementary Table S4, where class-I accounts for 69%, Class II 25% and Unknown 2%. The above data all indicate that *D. Salina* genome is a highly repetitive genome (Supplementary Table S4). 77,287 ESTs were constructed by means of transcriptome analysis without parameter assembly, and 96.21% of the ESTs were covered by the EST database. The results of comparison were shown in Supplementary Table S5, indicating that the integrity of the genome coding region was high.

**Figure 2:**
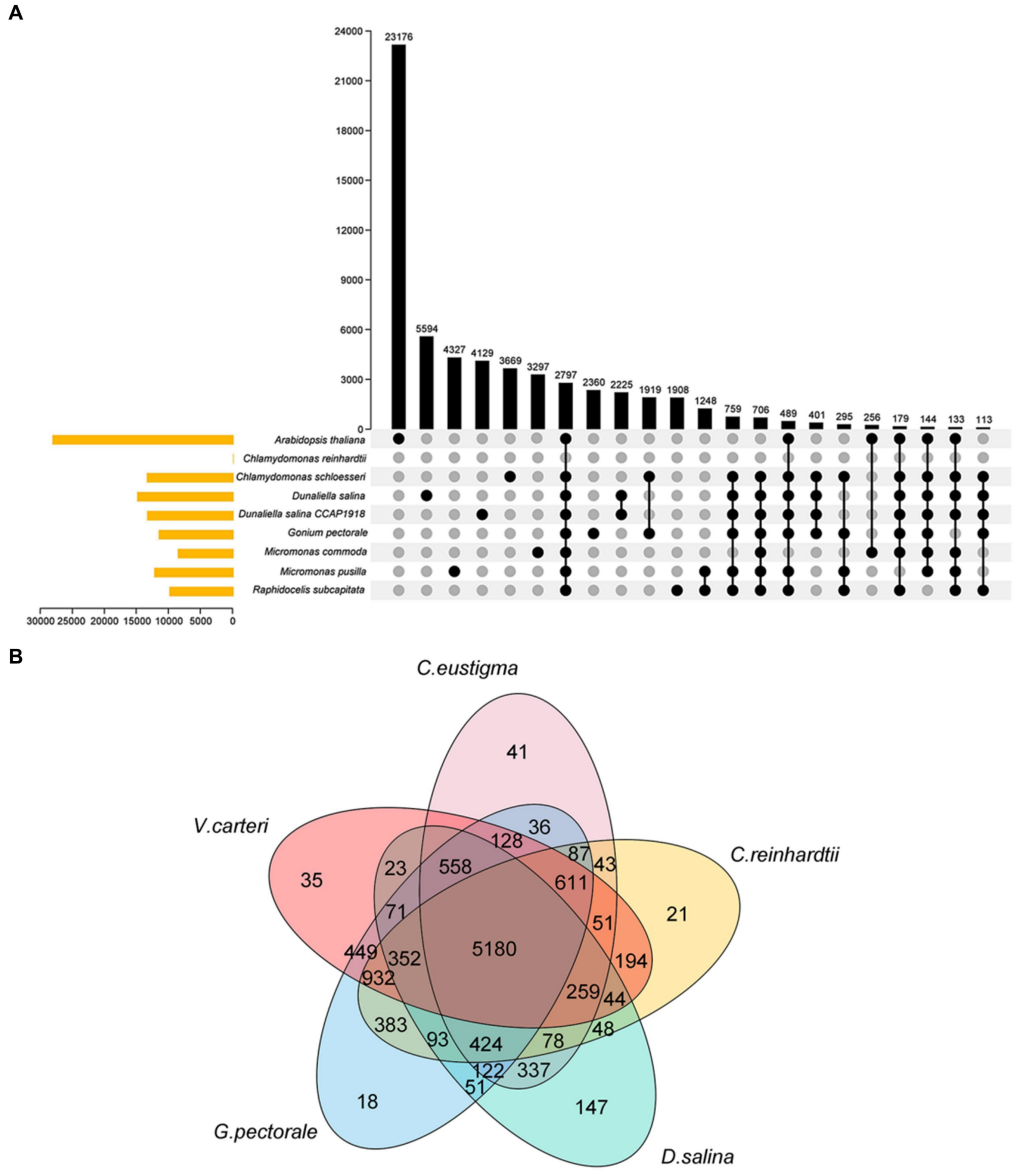
Venn diagram of the gene family. **(A)** Venn diagram of gene families of 9 algae species. **(B)** Venn diagram of gene families of 5 algae species.

*C. eustigma*is, an acidophilic extremophile, is also an extreme organism. Both *C. eustigma*is and *D. salina* are representative of extremophiles within the Volvocales order (Hirooka et al., 2017). *G.pectoral* and *V. carteri* are representative multicellular taxa in Volvocales (Prochnik et al., 2010; Hanschen et al., 2016). Using *C. rheinhardtii* as a neutrophilic relative reference for polar organisms, and based on current DNA sequencing analysis, it is the most likely ancestral species that evolved from single-celled to multicellular groups. So we selected these most representative species for our next comparative genomics analysis (Merchant et al., 2007), *G. pectorale* and *V. carteri*, along with the current genome, enabled us to analyze the evolutionary relationships of species within the Volvocales family using OrthoFinder software (Figure 2A) (Emms and Kelly, 2015). This provides a reference for inferring the genome content and structure of the most recent common ancestor of all extant Volvocales, thereby improving our understanding of their origins and evolution. The results showed that 10,934 gene families were clustered, including 1,020 single-copy orthologous gene families (Figure 2A). After extracting the gene families of each species, the total number of gene families for the five species was 5,180, with 41 (0.4%) specific to *C. eustigm*a, 21 (0.2%) specific to *C. reinhardtii*, 35 (0.3%) specific to *V. carteri*, 18 (0.2%) specific to *G. pectorale*, and 147 (1.3%) specific to *D. salina* (Figure 2B).

**Figure 3:**
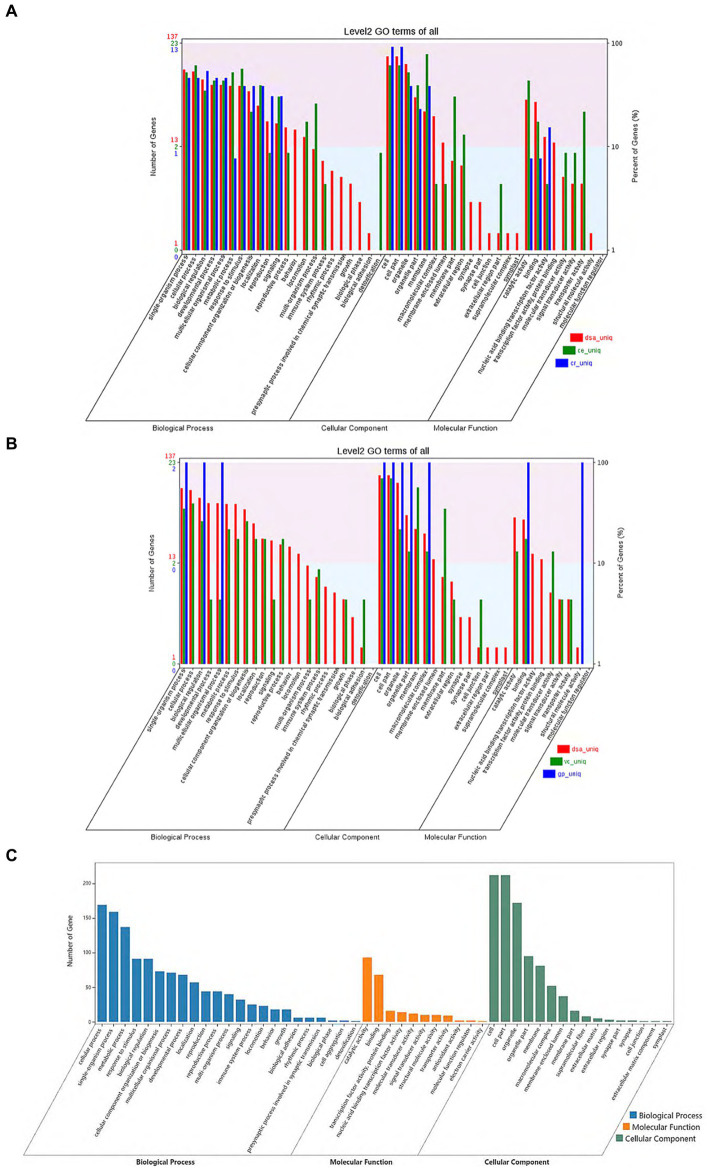
Comparison of functional annotation of specific and common gene families. **(A)** Comparison of functional annotation of specific gene families in *D. salina, C. eustigma, C. reinhardtii*. **(B)** Comparison of functional annotation of specific gene families in *D. salina, Vcarteri, G. pectoral*. **(C)** Comparison of functional annotation of the common gene families in *D. salina, C. eustigma*.

From the gene family function annotation results and considering the data in Figures 3A,B, all gene family functions in *D. salina* are primarily concentrated in cellular processes, metabolic processes, multicellular organismal processes, catalytic reactions in molecular functions, membrane in cellular components, and cell parts in cellular components within the biological process when compared to the other four species. A comparison of functional annotation of specific gene families in *D. salina, V. carteri, and G. pectoral* was conducted. Since both *D. salina* and *C. eustigma* are extreme organisms, with *D. salina* being a halophilic microorganism and *C. eustigma*, an acidotolerant microorganism, we aimed to explore the function of gene families shared by extreme organisms in Volvocales. To achieve this, we performed GO functional enrichment. During biochemical processing, the highest number of gene families were enriched by cellular processes, single-organism processes, and metabolic processes. This was followed by response to stimulus, biological regulation, and detoxification. In comparison to molecular functional enrichment, the number of gene families enriched in molecular functions was significantly smaller, mainly including catalytic activity, binding, and antioxidant activity. Regarding cellular components, the primary focus was on cells, cell components, organelles, organelle parts, and membranes. These functions facilitate the normal growth of extreme organisms in their corresponding environments (Figures 3A–C).

### Evolution and phylogeny of the *Dunaliella salina* genome

**Figure 4:**
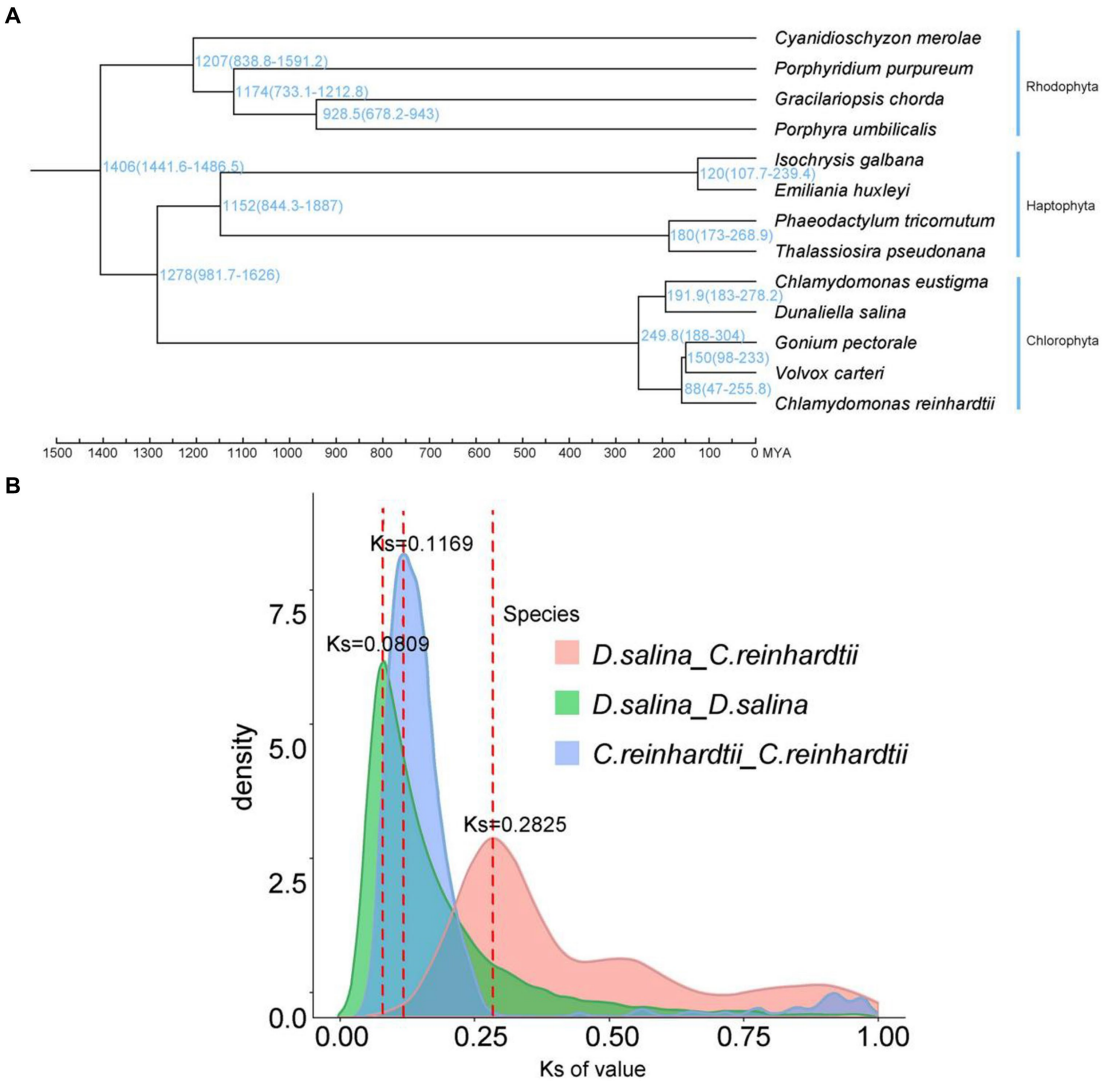
Characterization and comparative analysis of the *D. salina* genome. **(A)** Phylogenetic tree and divergence times of *D. salina*. **(B)** Density distribution of *D. salina* and *C. reinhardti*.

We constructed a high-confidence phylogenetic tree and estimated the divergence times of the 13 species using genes extracted from a total of 1,020 single-copy families. The results of species evolutionary tree clustering showed one branch of the multicellular group, where the two extremophiles clustered together, further demonstrating that the two representative multicellular algae, *G. pectorale* and *V. carteri*, evolved from the ancestral species of *C. reinhardtii*, and differentiated later than *D. salina* and *C. eustigma*. The expansion and contraction of gene families allow us to observe the changes in gene families during the evolution of species. We discovered 79 expanded and 5 contracted gene families in *D. salina* (Figure 4A).

According to the syntenic blocks detected in *C. eustigma, C. reinhardtii, D. salina, G. pectorale and V. carteri*. The *D. salina* whole-genome duplication (WGD) took place 13 MYA (Ks ≈ 0.0809) and *C. reinhardtii* whole-genome duplication (WGD) that took place 19 MYA (Ks ≈ 0.1169) (Figure 4B). We construct a phylogenetic tree. In combination with R8s software, the reported fossil evidence of these three species is *C. reinhardtii* (2.5 million years), *G. pectorale* (1.5 million years) and *V. carteri* (65–99 million years). Volvocales *C. eustigma*, *D. salina*, and other species diverged about 250 million years ago, *C. eustigma* and multicellular algae diverged about 232.5 million years ago, and *G. pectorale* and *Volvox* diverged about 150 million years ago (Figure 4A). Chlorophyta and haphtophyta diverged about 1,278 million years ago. And rhodophyta diverged with the chlorophyta and haphtophyta about 1,406 million years ago.

We used synonymous substitution rates (Ks) between collinear paralogous genes to identify potential whole-genome duplication (WGD) events, based on the assumption that the number of silent substitutions per site between two homologous sequences increases in a relatively linear manner with time. A density plot of Ks values for the collinear gene pairs of *D. salina* and *C. reinhardtii* suggested a more recent genome-wide replication event between *D. salina* and *C. reinhardtii* occurred about 46 million years ago, and an earlier genome-wide replication event 122 million years ago. The *D. salina* genome had a large-scale gene replication event about 1.3 million years ago, while the *C. reinhardtii* genome had a large-scale gene replication event about 1.9 million years ago (Figure 4B).

### Analysis of the transcriptome involved in β-carotene content in *Dunaliella salina* FACHB-435 under high light stress

To further investigate the mechanism of β-carotene accumulation in *D. salina* under high light stress, this study analyzed the differences in gene expression in the carotenoid metabolic pathway of *D. salina* under high light stress and normal light incubation using high-throughput transcriptome sequencing. The evaluation of the sequencing output data for the seven samples during high light stress of *D. salina* is shown in the Supplementary Table S6.

**Figure 5:**
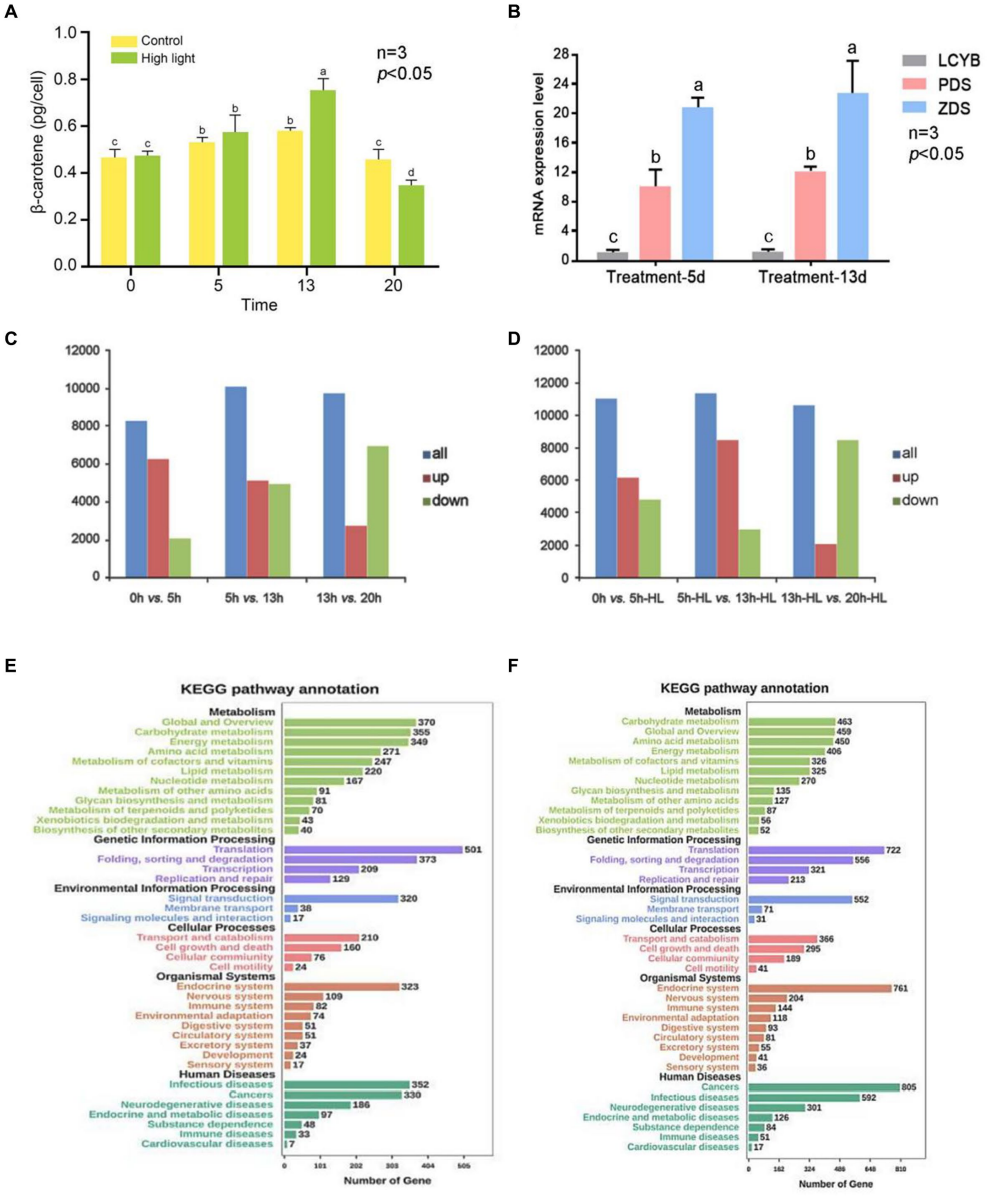
Overview of transcriptional analysis of *D. salina* under different light intensity. **(A)** Levels of β-carotene in *D. salina* across various growth phases when exposed to high and normal light intensities. **(B)** The verification of qRT-PCR. **(C)** The statistical results of DEGs in the control group. **(D)** The statistical results of DEGs in the high-light treatment group. **(E)** KEGG-enriched analysis of differentially expressed genes in the control group. **(F)** KEGG-enriched analysis of differentially expressed genes in the high-light treatment group.

The percentage of clean reads was high, indicating good sequencing quality. Additionally, the GC content of the bases in all seven libraries was between 50 and 60%, which is within the normal range. The Figure 5A shows the accumulation of β-carotene in single algal cells of *D. salina* under high light and control conditions. The accumulation of β-carotene in the high light and control groups increased and then decreased with the increase of incubation time. The β-carotene content in the high light experimental group was significantly higher than that in the control group at day 13 (*p* < 0.05). Afterward, it gradually became lower than that of the control group. The results showed that high light could promote the accumulation of β-carotene in *D. salina* at a certain stage of culture (Figure 5A).

The annotation success rates for the four major databases are shown in Supplementary Table S7. The percentage of unigenes successfully annotated in eggNOG is 61.87%, in GO database is 34.31%, in KOG database is 61.87%, and in KEGG database is 21.30%. These percentages indicate that the gene function annotation of the transcriptome is comprehensive (Supplementary Table S7).

As shown in Supplementary Figure S2 and Supplementary Tables S7, S8, GO categories include: A total of 35,230 unigene notes were classified into cell component classification, among which the largest proportion of unigene related to cells and cell components accounted for 22.7%, and the unigene related to organs and organ components accounted for 29.4%. These categories make up the majority of cell classification notes. A total of 32,412 unigene annotations were classified into the classification of biological processes, of which 12.6% were related to metabolic processes, 14.2% were related to cellular biological processes, and 13.5% were related to single biological processes. These categories make up the majority of cell metabolism annotations. A total of 628 unigene annotations were classified into the classification of molecular processes, among which binding related unigene accounted for 29.6% and catalytic activity related unigene accounted for 43.9%, which accounted for the majority of molecular process annotations. In summary, most of the genes in the classification of cell components were related to organelles, cell membranes and macromolecular complexes. In the process of biological metabolism, most genes are related to stress response, signal transduction pathway and metabolic response. In terms of molecular function, most genes are related to transport, catalysis and binding activities (Supplementary Figure S3 and Supplementary Tables S7, S8).

KOG was divided into 26 annotation groups, and the genes successfully annotated by KOG were classified according to the KOG group. The results were shown in Supplementary Table S9, as shown in Supplementary Figure S3. A total of 7,955 unigene were annotated in the KOG classification. Among them, the highest gene annotation included general function prediction (13%), signal transduction mechanism (10%), and post-translational modification (11%). 274 genes related to lipid transport and metabolism were found in KOG annotation. The above data indicated that in the early stage of stress, a large number of algal cells carried out biological processes such as transcriptional activation and protein translation in response to stress, accompanied by lipid anabolism (Supplementary Figure S4 and Supplementary Table S9).

DEGs between two adjacent time points **(AVsB)** of stress treatment group and control group were detected by DEGseq software, and DEGs were divided into up-regulated and down-regulated genes. The statistical results of DEGs in the high-light treatment group and control group are shown in Supplementary Table S10 and Figures 5C,D. The results showed that the gene expression of *D. salina* under high light stress was different from that of normal culture. The most obvious difference was that in the control group, 6,142 up-regulated genes were lower than 0dVs5d (6250), while 3,949 down-regulated genes were higher than 0dVs5d (2068) in 5dVs13d differential genes (Figures 5C,D; Supplementary Table S10).

In the high-light treatment group, the number of up-regulated genes in 5d-HLVs13d-HL was 8,466, higher than that of 0dVs5d-HL (6123) in the previous stage, while the number of down-regulated genes was 2,969, lower than that of 0dVs5d-HL (4886). At the same time, it was also observed that compared with the previous stage of 13dVs20d and 13d-HLVs20d-HL, the number of up-regulated genes decreased and the number of down-regulated genes increased, and the number of down-regulated genes was more than the number of up-regulated genes. According to the data analysis, the transcriptome of *D. salina* underwent drastic changes in the early stage, and the transcriptome was in an activated state, and then showed a downward trend, and the number of down-regulated genes in each differentially expressed gene set was lower than that of up-expressed genes, except for 13-20d. The number of DEG in the treatment group was higher than that in the control group, and the number of genes up-regulated by 5d-HLVs13d-HL was relatively higher, and the number of genes up-regulated by 20d-HL was at least 2,101. It can be seen from the above that the number of differential genes before and after 5 to 13 days of culture was the highest in both high-light stress and normal culture of *D. salina*, and the number of up-regulated genes was higher than that of down-regulated genes (Supplementary Table S10).

Through the enrichment of differentially expressed genes in the KEGG pathway, 52 genes in the metabolic pathway were annotated in the biosynthesis of secondary metabolites, 325 genes were annotated in lipid metabolism, and 87 genes were annotated in the metabolism of terpenes and polykeones. KEGG enrichment of differential genes is shown in Figure 5E. The predicted up-regulated and down-regulated gene enrichment numbers of KEGG pathway are shown in Supplementary Tables S11, S12. It notes that the predicted up-regulated genes of carotenoid metabolic pathway include *DsCrtB, DsPDS, DsZ-ISO, DsZDS, DsCRTISO, DsLUT5, DsCrtL-B* and *DsCCD8*, while the predicted down-regulated genes include *DsCrtF* and *DsLUT1*. The four genes that were both up-regulated and down-regulated were *DsZEP, DsCrtR-b, DsCruA/P* and *DsCrtZ 4* (Supplementary Table S13).

Through the enrichment of 5d Vs13d differentially expressed genes in KEGG pathway, 40 genes in metabolic pathway were annotated in secondary metabolite biosynthesis, 220 genes were annotated in lipid metabolism, and 70 genes were annotated in terpenoid and polyketo metabolism. The down-regulation of carotenoid metabolic pathway prediction genes is shown in Supplementary Table S14. There are 12 down-regulated genes, namely *DsCrtB, DsPDS, DsZ-ISO, DsZDS, DsCrtISO, DsLUT5, DsCrtF, DsLUT1, DsZEP, DsCrtR-b, DsCruA/P* and *DsZEP*. *DsCrtL-b* and *DsCCD8* are both upward and downward forecast.

KEGG enrichment of differential genes is shown in Figure 5F. The predicted up-regulated and down-regulated gene enrichment numbers of KEGG pathway are shown in Supplementary Tables S15, S16. It can be seen from the results that compared with the results of 5d-HLVs13d-HL, the genes enriched by 5D-VS13D KEGG differential genes in the carotenoid metabolic pathway were mostly down-regulated genes, which may be closely related to the culture under high light stress condition. Moreover, on the 13th day of culture, the β-carotene content in the high light group was significantly higher than that in the control group (Supplementary Tables S15, S16). It was further indicated that the high light stress could promote the synthesis of carotene in the salt algae.

### RT-PCR verification

When comparing data from 5 days versus 13 days, the genes expressed differently in the carotenoid metabolism pathway during the 5-day high light versus 13-day high light comparison were predominantly up-regulated, leading to increased β-carotene levels in individual cells compared to the control. This further supports the conclusion that high light stress enhances β-carotene synthesis. As shown in the Figure 5B, the expression of *DsPDS* and *DsZDS* genes was higher on day 13 under high light treatment than on day 5. The expression of both *DsPDS* and *DsZDS* genes increased on day 13 compared to day 5, which was consistent with the predicted results. The β-carotene accumulation of individual cells in the high light experimental group and the control group was compared on day 5 and day 13. The β-carotene content of single cells was higher in the high light group than in the control group on day 13, which was consistent with the statistical results of differentially expressed genes. Since the *DsLCYB* gene is one of the key enzymes for β-carotene synthesis, and there was no *DsLCYB* gene among the predicted up-and down-regulated genes, the expression of the *DsLCYB* gene was verified. The results showed that there was no significant change in the expression of the *DsLCYB* gene in the samples from the two time periods, which was consistent with the predicted results (Figure 5B).

## Discussion

We sequenced the whole genome of *D. salina* FACHB-435 and annotated the assembly. The genome was assembled to the scaffold level. The quality of genome assembly is higher compared with the published *D. salina* CCAP19/18 genome (Polle et al., 2017) (Supplementary Table S17). According to the results of the genome assessment, *D. salina* FACHB-435 genome has a 472 Mb genome size with a contig N50 of 458 Kb, while *D. salina* CCAP19/18 genome 343 Mb genome size with a contig N50 of 343 K. The genome size of *D. salina* was significantly different among different strains, suggesting that there was large variation and rich diversity of genome size in this species. Therefore, the characteristics of secondary metabolites synthesized by different *D. salina* strains are also diverse, which is advantageous for us to screen high-quality strains from different strains to meet the needs of industrialization. A total of 30,752 protein-coding genes were predicted in *D. salina* FACHB-435. The annotation results evaluated by BUSCO was shown that completeness was 91.0% and replication was 53.1%. The fragments were 6.3% and the deletions were 2.6%. In *D. salina* CCAP19/18, 16,697 protein-coding genes were predicted (Supplementary Table S17). The difference in results may be caused by the loss of some lineal homologous gene sets, and the difference in sequencing and assembly methods. At present, our annotated genome is the most complete and can be used for the related study of *D. salina*.

Comparative genomics was used to analyze the evolution of genomes in Volvox order. The results revealed the evolutionary relationships among *Chlamydomonas elegans, Chlamydomonas Rhine, Dunaliella salina, Discoplecta pectoralis* and *Volvox*. It revealed that *A. thaliana* diverged from Volvocales about 448 million years ago, then *Volvocales C. eustigma*, *D. salina*, and other species diverged about 250 million years ago. In about 136 million years, *C. elegans* and *D. Salina* had a genome-wide replication event, which may have been the time node for the differentiation of these two species. Combined with the changes of geographical environment, the earth’s climate has been in the alternating changes of cold and warm from the birth of the earth to the existence of life activities. Abiotic factors such as sea-land drift caused five mass extinctions of species living on Earth. However, *C. elegans* and *D. salina* occurred genome-wide replication events in the Jurassic period (100 million years ago), when the species themselves mutated (large-scale gene replication), in which the progeny suitable for survival were selected and retained by the changing environment at that time, and the genetic information was steadily continued. Multicellular species such as Pectoralis and Volvox evolved from single-celled algae such as *C. rheinacea*. The species tree was constructed with a single copy of the orthologous gene, and the evolutionary time of these species in Volvox order was deduced by combining the fossil evidence of the species. The results show that multicellular algae were preserved by a certain stage of multicellular transformation of *C. rheinacea*, and developed into modern multicellular algae such as Thoracodiscus and Volvox. *D.salina* has the characteristic of halophilia, which makes it a kind of extreme organism. The functional enrichment of its unique gene family may be related to the structure of flagella, cell membrane and halophilia. Specific localization of a gene or a metabolic process requires further research and analysis to locate the halophile characteristics to certain genes. These genetic resources are expected to be applied in the industrialization practice of carotenoid production by *D. Salina*.

Studies have shown that *D. salina* can accumulate a large amount of beta-carotene under stress. It has been reported that the β-carotene content of *D. salina* can reach 14% under certain conditions (Ye et al., 2008). However, under inappropriate conditions, the β-carotene content of *D. salina* is 0.3% or less (Johnson and Schroeder, 1996). Salinity can also promote the accumulation of β-carotene in microalgae. Effects of *Chlamydomonas* sp. ICE-L on growth, lipid yield and fatty acid desaturase gene expression under NaCl stress was analyzed. With the gradual increase of NaCl concentration, the growth rate of *Chlamydomonas* gradually decreased, when NaCl was 16‰, the lipid content reached the highest, reaching 23% (w/w) (An et al., 2013). Light intensity is an important factor affecting the accumulation of carotene in algae. Appropriate light intensity can promote the synthesis of carotene, but too strong light will inhibit the synthesis of carotene. Studies have shown that light intensity has a significant impact on algal pigment synthesis and accumulation.

High light stress can promote the synthesis of carotene in *D. salina* to a certain extent. Under different light intensity culture, the higher the light intensity, the faster the cell growth. Our experiment found that the accumulation of β-carotene in FACHB-435 single algal cells of *D. salina* under high light and control conditions, with the increase of culture time, The β-carotene content of the high-light experimental group and the control group increased first and then decreased, and the β-carotene content of the high-light experimental group was significantly higher than that of the control group on the 13th day of culture (*p* < 0.05), an increase of 30.3%. However, we noted that high light stress made *D. salina* more likely to enter the decline stage, the algal cells gradually turned yellow. Though the accumulation of β-carotene in single cells was higher than that in the control group at the early stage of culture, was lower than that in the control group after the 13th day. Therefore, in order to obtain the best induction effect, attention should be paid to controlling induction conditions, especially the length of induction time, in order to induce carotenoid synthesis by high light stress.

In this study, we compared the gene expression profile of *D. salina* under different temperatures and light intensity by using transcriptomic methods, and revealed its molecular mechanism of adapting to stress. The differentially expressed genes between two adjacent time points of the stress experimental group and the control group were compared, and the single-cell β-carotene accumulation of the experimental group and the control group was investigated. Other researchers also found that β-carotene accumulation increases under bright light conditions, and transcriptional activation of β-carotene biosynthesis genes during the initial phase of stress exposure is the determining factor for increased beta-carotene accumulation in microalgae, which helps microalgae survive in harsh environments (Zhu et al., 2020). Another study found that significant beta-carotene accumulation after irradiation of 400 μmol photons ·m − 2·s − 1 for at least 8 h (Xi et al., 2021). The results showed that the β-carotene accumulation of the treated group was higher than that of the control group. It is consistent with the down-regulation trend of differential genes. High light could promote the accumulation of β-carotene in *D. salina* at a 13 d stage of culture. The enrichment of DEGs in KEGG, it notes that the predicted up-regulated genes of carotenoid metabolic pathway include *DsCrtB, DsPDS, DsZ-ISO, DsZDS, DsCRTISO, DsLUT5, DsCrtL-B* and *DsCCD8*, while the predicted down-regulated genes include *DsCrtF* and *DsLUT1*. The four genes that were both up-regulated and down-regulated were *DsZEP, DsCrtR-b, DsCruA/P* and *DsCrtZ 4*.

Among the up-regulated genes, *CrtB* is 15-cis-phytoene synthase, which catalyzes GGPP to generate 15-cis-phytoene. *PDS* is a 15-cis-phytoene desaturase that catalyzes 15-cis-phytoene to produce zta-carotene and 9,15,9 ‘-Tricis-zta-carotene. It is then catalyzed by *Z-ISO* (zeta-carotene isomerase), *ZDS* (zeta-carotene desaturase) and *CRTISO* (prolycopene isomerase) to produce all-trans-Lycopene. Gamma-carotene was produced from all-trans-Lycopene by lycopene beta-cyclase (*lcyB, crtL1, crtY*). Gamma-carotene was catalyzed to beta-carotene by lycopene beta-cyclase(*lcyB, crtL1, crtY, cruA, cruP*). We hypothesized that up-regulation of these genes could promote the increase of β-carotene content. Synthesis of β-carotene could be promoted by regulating the high expression of these genes. Among the down-regulated genes, *CrtF* is demethylspheroidene O-methyltransferase, a gene on the all-trans-Neurosporene bypass, and *LUT1* is a gene on the all-trans-Lycopene bypass. Down-regulation of these two genes was beneficial to regulate metabolic flow towards β-carotene synthesis. The content of β-carotene could be increased by interfering with their expression.

Three differentially expressed genes were selected for RT-PCR verification. The expression levels of *DsPDS* and *DsZDS* genes in the experimental group increased on day 13 compared with day 5, which was consistent with the prediction, while the expression levels of *DsLCYB* genes had no significant change. These studies have revealed that light intensity has a great effect on the growth and β-carotene accumulation of *D. salina*, and the results have important significance for further understanding the β-carotene synthesis mechanism of *D. salina*. We found that the expression of some important functional genes in the carotenoid synthesis pathway is changed due to high light stress, and these genes can be used as nodes to regulate carotenoid synthesis. In the future, overexpression and interference of these genes can be carried out to study their regulatory effects on carotenoid synthesis.

Zamani and Moradshahi (2016) explored the differences in transcription levels of the *DsPDS* gene under high light conditions. They tested the transcriptional expression of the *DsPDS* gene when exposed to high light for 3, 6, 12, 24 h. The results showed that before 12 h of high light treatment, the mRNA expression of the *DsPDS* gene in the treatment group was higher than in the control group. At 12 h, the mRNA expression in the treatment group was 2.2 times that of the control group. However, after 24 h of exposure, the *DsPDS* mRNA expression in the treatment group dropped and was lower than the control group. Said Rabbani et al. showed that under high light conditions (690 μmol m-2 s-1), the expression of *DbPSY* and *DbPDS* genes in *D. bardawil* did not increase. Different *Dunaliella* strains adapt differently to stress conditions, and the mechanism of β-carotene accumulation in *D. salina* under high light stress needs further exploration. According to the experimental results of Rabbani et al. (1998), the expression of PSY and PDS genes was not up-regulated under the condition of high light (690 μmol m-2 s-1). These results imply that the adaptation of different *Dunaliella* strains to stress conditions is different. The mechanism of carrot accumulation in *D. salina* under high light stress needs further investigation in future.

This research delves into the genomics and transcriptomics of *D. salina* to uncover the synthesis mechanism of β-carotene, a crucial compound for pharmaceutical formulations and other industries. The study successfully sequenced the *D. salina* FACHB-435 genome, revealing insights into the carotenoid metabolic pathway and identifying key genes that regulate β-carotene synthesis. These findings are vital for enhancing β-carotene production in *D. salina*, with implications for improving the efficiency and sustainability of industrial practices in pharmaceutical applications. By manipulating light conditions and understanding the genetic underpinnings of β-carotene accumulation, this research paves the way for optimized cultivation strategies, potentially revolutionizing the industrial application of microalgae.

## Data availability statement

The original contributions presented in the study are included in the article/Supplementary material, further inquiries can be directed to the corresponding authors.

## Author contributions

DC: Writing – review & editing, Writing – original draft, Visualization, Validation, Supervision, Software, Resources, Project administration, Methodology, Investigation, Funding acquisition, Formal analysis, Data curation, Conceptualization. ZL: Writing – original draft, Methodology, Investigation, Formal analysis, Data curation. JS: Writing – original draft, Methodology, Investigation, Formal analysis, Data curation. HS: Writing – review & editing, Investigation, Formal analysis, Data curation. XZ: Writing – original draft, Software, Resources, Methodology, Investigation, Formal analysis, Data curation. CZ: Writing – original draft, Methodology, Investigation, Formal analysis. YC: Writing – review & editing, Writing – original draft, Supervision, Methodology, Investigation, Formal analysis. TX: Writing – review & editing, Writing – original draft, Visualization, Validation, Supervision, Software, Resources, Project administration, Methodology, Investigation, Funding acquisition, Formal analysis, Data curation, Conceptualization.
