# Supplementary material for: Genomics and transcriptomics reveal β-carotene synthesis mechanism in Dunaliella salina
Source: Front Microbiol. 2024 May 17;15:1389224. doi: 10.3389/fmicb.2024.1389224 (PMC11140103; doi:10.3389/fmicb.2024.1389224)
Supplement: Supplementary file 1 [file Data_Sheet_1.zip › Supplementary Tables.docx]

Table S1 Geneome survey statistics of of *Dunaliella Salina* FACHB-435

| Sample | KmerSize | HeterZygosity | GenomeLength | RepeatLength | UniqLength |
| --- | --- | --- | --- | --- | --- |
| *Dunaliella Salina* FACHB-435 | 17 | 0.287% | 386,492,685 | 105,329,514 | 281,163,171 |

Table S2 Statistics of the completeness of the assembled genomes for FACHB-435 by BUSCO

| Description | Number | Percentage (%) |
| --- | --- | --- |
| Complete BUSCOs (C) | 276 | 91 |
| Complete and single-copy BUSCOs (S) | 128 | 42.2 |
| Complete and duplicated BUSCOs (D) | 148 | 48.8 |
| Fragmented BUSCOs (F) | 19 | 6.3 |
| Missing BUSCOs (M) | 8 | 2.7 |
| Total BUSCO groups searched | 303 | 100 |

Table. S3 BUSCO assesses genome integrity

| **Description** | **Number** | **Percentage (%)** |
| --- | --- | --- |
| Complete BUSCOs (C) | 225 | 74.2 |
| Complete and single-copy BUSCOs (S) | 84 | 27.7 |
| Complete and duplicated BUSCOs (D) | 141 | 46.5 |
| Fragmented BUSCOs (F) | 10 | 3.3 |
| Missing BUSCOs (M) | 68 | 22.5 |
| Total BUSCO groups searched | 303 | 100 |

Table. S4 Repetitive sequence classification

|  | **Number** | **Length (Mb)** | **% of repeats** | **% of genome** |
| --- | --- | --- | --- | --- |
| **Total repeat fraction** | 919625 | 206325526 | 100 | 43.75 |
| **Class I: Retroelement** | 410968 | 142648034 | 69.14 | 30.25 |
| **LTR Retrotransposon** | 92313 | 24853950 | 12.05 | 5.27 |
| Ty1/Copia | 2106 | 1575470 | 0.76 | 0.33 |
| Ty3/Gypsy | 10400 | 7519687 | 3.64 | 1.59 |
| Other | 79807 | 15758793 | 7.64 | 3.34 |
| **Non-LTR Retrotransposon** | 267780 | 107139205 | 51.93 | 22.72 |
| LINE | 249115 | 104763280 | 50.78 | 22.21 |
| SINE | 18665 | 2375925 | 1.15 | 0.5 |
| **Unclassified retroelement** | 50875 | 10654879 | 5.16 | 2.26 |
| **Class II: DNA transposon** | 193121 | 50870461 | 24.66 | 10.79 |
| **TIR** |  |  |  |  |
| CMC [DTC] | 12763 | 3153384 | 1.53 | 0.67 |
| hAT | 3370 | 1530315 | 0.74 | 0.32 |
| Mutator | 806 | 969077 | 0.47 | 0.21 |
| Tc1/Mariner | 90 | 16216 | 0.01 | 0 |
| PIF/Harbinger | 183 | 74767 | 0.04 | 0.02 |
| Other | 175819 | 45110486 | 21.86 | 9.57 |
| **Helitron** | 376 | 91353 | 0.04 | 0 |
| **Tandem Repeats** | 380177 | 25838183 | 12.52 | 5.48 |
| **Unknown** | 11516 | 4212909 | 2.04 | 0.89 |

Table S5 Statistics of the completeness of the protein for FACHB-435 by BUSCO

| **Description** | **Number** | **Percentage (%)** |
| --- | --- | --- |
| Complete BUSCOs (C) | 276 | 91.1 |
| Complete and single-copy BUSCOs(S) | 115 | 38.0 |
| Complete and duplicated BUSCOs (D) | 161 | 53.1 |
| Fragmented BUSCOs (F) | 19 | 6.3 |
| Missing BUSCOs (M) | 8 | 2.6 |
| Total BUSCO groups searched | 303 | 100 |

Table S6 Sequencing quality statistics of seven samples

| Sample | %Aligned | Total raw  reads | Total clean  reads | Total raw  bases | Total clean  bases | %Duplication | GC content | %PF |
| --- | --- | --- | --- | --- | --- | --- | --- | --- |
| 0d | 92.40% | 65923340 | 9736084779 | 9888501000 | 9736084779 | 16.40% | 55.87% | 14769.53% |
| 5d | 93.37% | 63109094 | 9328956748 | 9466364100 | 9328956748 | 15.40% | 56.30% | 14783.73% |
| 13d | 93.10% | 74160390 | 11000391861 | 11124058500 | 11000391861 | 13.83% | 56.43% | 14829.20% |
| 20d | 93.10% | 63731592 | 9438045716 | 9559738800 | 9438045716 | 15.27% | 56.20% | 14808.37% |
| 5d-HL | 93.23% | 68030186 | 8598187334 | 8704527900 | 8598187334 | 14.83% | 56.10% | 14816.70% |
| 13d-HL | 92.87% | 74584348 | 11065565614 | 11187652200 | 11065565614 | 15.87% | 55.90% | 14835.63% |
| 20d-HL | 92.80% | 72327404 | 10731777923 | 10849110600 | 10731777923 | 13.47% | 55.97% | 14832.97% |

Table S7 The statistics of gene annotation success rate

| database | number | percentage |
| --- | --- | --- |
| EggNOG | 16,303 | 61.87% |
| GO | 9,040 | 34.31% |
| KOG | 16,303 | 61.87% |
| KEGG Pathway | 5,612 | 21.30% |

Table S8 Classified statistical chart of GO

| GO ID (level1) | GO Term (level1) | GO Term (level2) | Gene Number |
| --- | --- | --- | --- |
| GO:0005575 | cellular_component | cell part | 8004 |
| GO:0005575 | cellular_component | cell | 8004 |
| GO:0005575 | cellular_component | organelle | 6680 |
| GO:0008150 | biological_process | cellular process | 4866 |
| GO:0008150 | biological_process | single-organism process | 4698 |
| GO:0008150 | biological_process | metabolic process | 4098 |
| GO:0005575 | cellular_component | organelle part | 4059 |
| GO:0005575 | cellular_component | membrane | 3021 |
| GO:0008150 | biological_process | response to stimulus | 2833 |
| GO:0003674 | molecular_function | catalytic activity | 2763 |
| GO:0008150 | biological_process | biological regulation | 2473 |
| GO:0008150 | biological_process | developmental process | 2264 |
| GO:0008150 | biological_process | multicellular organismal process | 2187 |
| GO:0008150 | biological_process | cellular component organization or  biogenesis | 1905 |
| GO:0003674 | molecular_function | binding | 1858 |
| GO:0005575 | cellular_component | macromolecular complex | 1718 |
| GO:0008150 | biological_process | localization | 1312 |
| GO:0005575 | cellular_component | membrane-enclosed lumen | 1291 |
| GO:0008150 | biological_process | reproduction | 1184 |
| GO:0008150 | biological_process | reproductive process | 1089 |

Table S9 Classified statistical table of KOG

|  | Numbers | Class Name |
| --- | --- | --- |
| A | 679 | RNA processing and modification |
| B | 477 | Chromatin structure and dynamics |
| C | 604 | Energy production and conversion |
| D | 395 | Cell cycle control, cell division, chromosome partitioning |
| E | 511 | Amino acid transport and metabolism |
| F | 209 | Nucleotide transport and metabolism |
| G | 643 | Carbohydrate transport and metabolism |
| H | 357 | Coenzyme transport and metabolism |
| I | 427 | Lipid transport and metabolism |
| J | 790 | Translation, ribosomal structure and biogenesis |
| K | 1303 | Transcription |
| L | 582 | Replication, recombination and repair |
| M | 365 | Cell wall/membrane/envelope biogenesis |
| N | 10 | Cell motility |
| O | 1573 | Posttranslational modification, protein turnover, chaperones |
| P | 460 | Inorganic ion transport and metabolism |
| Q | 323 | Secondary metabolites biosynthesis, transport and catabolism |
| R | 0 | General function prediction only |
| S | 4675 | Function unknown |
| T | 1233 | Signal transduction mechanisms |
| U | 768 | Intracellular trafficking, secretion, and vesicular transport |
| V | 84 | Defense mechanisms |
| W | 116 | Extracellular structures |
| Y | 106 | Nuclear structure |
| Z | 498 | Cytoskeleton |
|  |  |  |

Table S10 Statistics on the number of differentially expressed genes between two adjacent time points

| differential expression genes | Number of differentially expressed genes | up-regulation | Down-regulation |
| --- | --- | --- | --- |
| 0dVs5d | 8318 | 6250 | 2101 |
| 5dVs13d | 8318 | 6142 | 3949 |
| 13dVs20d | 9766 | 2790 | 6976 |
| 0dVs5d-HL | 11009 | 6123 | 4886 |
| 5d-HLVs13d-HL | 11435 | 8466 | 2969 |
| 13d-HLVs20d-HL | 10563 | 2101 | 8462 |

Table S11 The enrichment number of predicted up-regulated gene in KEGG pathway (5d-HLVs13d-HL)

| KEGG_A_class | Pathway | out |
| --- | --- | --- |
| Genetic Information Processing | RNA transport | 94 |
| Metabolism | Biosynthesis of amino acids | 90 |
| Metabolism | Purine metabolism | 88 |
| Metabolism | Carbon metabolism | 81 |
| Metabolism | Pyrimidine metabolism | 76 |
| Metabolism | Oxidative phosphorylation | 43 |
| Genetic Information Processing | Basal transcription factors | 37 |
| Metabolism | Starch and sucrose metabolism | 36 |
| Metabolism | Pyruvate metabolism | 36 |
| Metabolism | Sphingolipid metabolism | 32 |
| Metabolism | Glycerophospholipid metabolism | 32 |
| Metabolism | Fatty acid metabolism | 31 |
| Metabolism | Photosynthesis | 30 |
| Metabolism | Carbon fixation in photosynthetic organisms | 29 |
| Metabolism | Nitrogen metabolism | 28 |

Table S12 The enrichment number of predicted down-regulated gene in KEGG pathway

(5d-HLVs13d-HL)

| **KEGG_A_class** | **Pathway** | **out** |
| --- | --- | --- |
| Metabolism | Pyrimidine metabolism | 80 |
| Metabolism | Oxidative phosphorylation | 72 |
| Metabolism | Glycolysis / Gluconeogenesis | 63 |
| Metabolism | Pyruvate metabolism | 59 |
| Metabolism | Porphyrin and chlorophyll metabolism | 58 |
| Metabolism | Fatty acid metabolism | 52 |
| Metabolism | Starch and sucrose metabolism | 47 |
| Metabolism | Glycerophospholipid metabolism | 47 |
| Metabolism | Carbon fixation in photosynthetic organisms | 44 |
| Metabolism | Citrate cycle (TCA cycle) | 43 |
| Metabolism | Glycerolipid metabolism | 42 |

Table S13 The differentially expressed genes of carotenoid pathway at 5d-HLVs13d-HL

| Classification of differentially expressed genes | genes |
| --- | --- |
| up-regulated genes | CrtB、PDS、Z-ISO、ZDS、CrtISO、LUT5、CrtL-B、CCD8 |
| down-regulated genes | CrtF、LUT1 |
| both up-regulated and down-regulated genes | ZEP、CrtR-b、CruA/P、CrtZ 4 |

Table S14 The differentially expressed genes of carotenoid pathway at 5dVs13d

| Classification of differentially expressed genes | genes | |
| --- | --- | --- |
| up-regulated genes | | 0 |
| down-regulated genes | | CrtB、PDS、Z-ISO、ZDS、CrtISO、LUT5、CrtF、LUT1、ZEP、CrtR-b、CruA/P、ZEP |
| both up-regulated and down-regulated genes | | CrtL-b、CCD8 |

Table S15 The enrichment number of predicted up-regulated gene in KEGG pathway

| **KEGG_A_class** | **Pathway** | **Count** |
| --- | --- | --- |
| Metabolism | Purine metabolism | 88 |
| Cellular Processes | Cell cycle | 81 |
| Genetic Information Processing | RNA transport | 72 |
| Metabolism | Biosynthesis of amino acids | 57 |
| Metabolism | Pyrimidine metabolism | 53 |
| Environmental Information Processing | Jak-STAT signaling pathway | 47 |
| Metabolism | Carbon metabolism | 44 |
| Metabolism | Sphingolipid metabolism | 36 |
| Metabolism | Glycerophospholipid metabolism | 30 |
| Genetic Information Processing | Basal transcription factors | 28 |
| Environmental Information Processing | Phosphatidylinositol signaling system | 26 |
| Metabolism | Inositol phosphate metabolism | 22 |
| Metabolism | Glycolysis / Gluconeogenesis | 21 |

Table S16 The enrichment number of predicted down-regulated gene in KEGG pathway

| **KEGG_A_class** | **Pathway** | **Count** |
| --- | --- | --- |
| Metabolism | Biosynthesis of amino acids | 175 |
| Metabolism | Purine metabolism | 133 |
| Metabolism | Oxidative phosphorylation | 104 |
| Metabolism | Pyrimidine metabolism | 103 |
| Metabolism | Pyruvate metabolism | 81 |
| Metabolism | Porphyrin and chlorophyll metabolism | 71 |
| Metabolism | Fatty acid metabolism | 69 |
| Metabolism | Starch and sucrose metabolism | 67 |
| Metabolism | Glycolysis / Gluconeogenesis | 67 |
| Metabolism | Carbon fixation in photosynthetic organisms | 63 |
| Metabolism | Photosynthesis | 59 |
| Metabolism | Glyoxylate and dicarboxylate metabolism | 57 |
| Metabolism | Citrate cycle (TCA cycle) | 52 |
| Metabolism | Glycerophospholipid metabolism | 49 |
| Metabolism | Fatty acid biosynthesis | 47 |
| Environmental Information Processing | PI3K-Akt signaling pathway | 44 |
| Metabolism | Glycerolipid metabolism | 41 |
| Metabolism | 2-Oxocarboxylic acid metabolism | 37 |
| Metabolism | Nitrogen metabolism | 36 |
| Metabolism | Sphingolipid metabolism | 36 |
| Metabolism | Pentose phosphate pathway | 35 |
| Metabolism | Terpenoid backbone biosynthesis | 34 |
| Metabolism | Carbon fixation pathways in prokaryotes | 33 |
| Environmental Information Processing | Phosphatidylinositol signaling system | 33 |
| Environmental Information Processing | Sphingolipid signaling pathway | 32 |
| Metabolism | N-Glycan biosynthesis | 29 |
| Metabolism | Pantothenate and CoA biosynthesis | 29 |
| Metabolism | Biosynthesis of unsaturated fatty acids | 27 |
| Genetic Information Processing | Basal transcription factors | 27 |
| Metabolism | Carotenoid biosynthesis | 25 |
| Metabolism | Ubiquinone and other terpenoid-quinone biosynthesis | 25 |
| Metabolism | Fatty acid degradation | 24 |
| Cellular Processes | Regulation of actin cytoskeleton | 24 |
| Metabolism | Inositol phosphate metabolism | 22 |
| Environmental Information Processing | Phospholipase D signaling pathway | 20 |
