# Supplementary material for: Genomics and transcriptomics reveal β-carotene synthesis mechanism in Dunaliella salina
Source: Front Microbiol. 2024 May 17;15:1389224. doi: 10.3389/fmicb.2024.1389224 (PMC11140103; doi:10.3389/fmicb.2024.1389224)
Supplement: Supplementary file 1 [file Data_Sheet_1.zip › Supplementary Figures.docx]

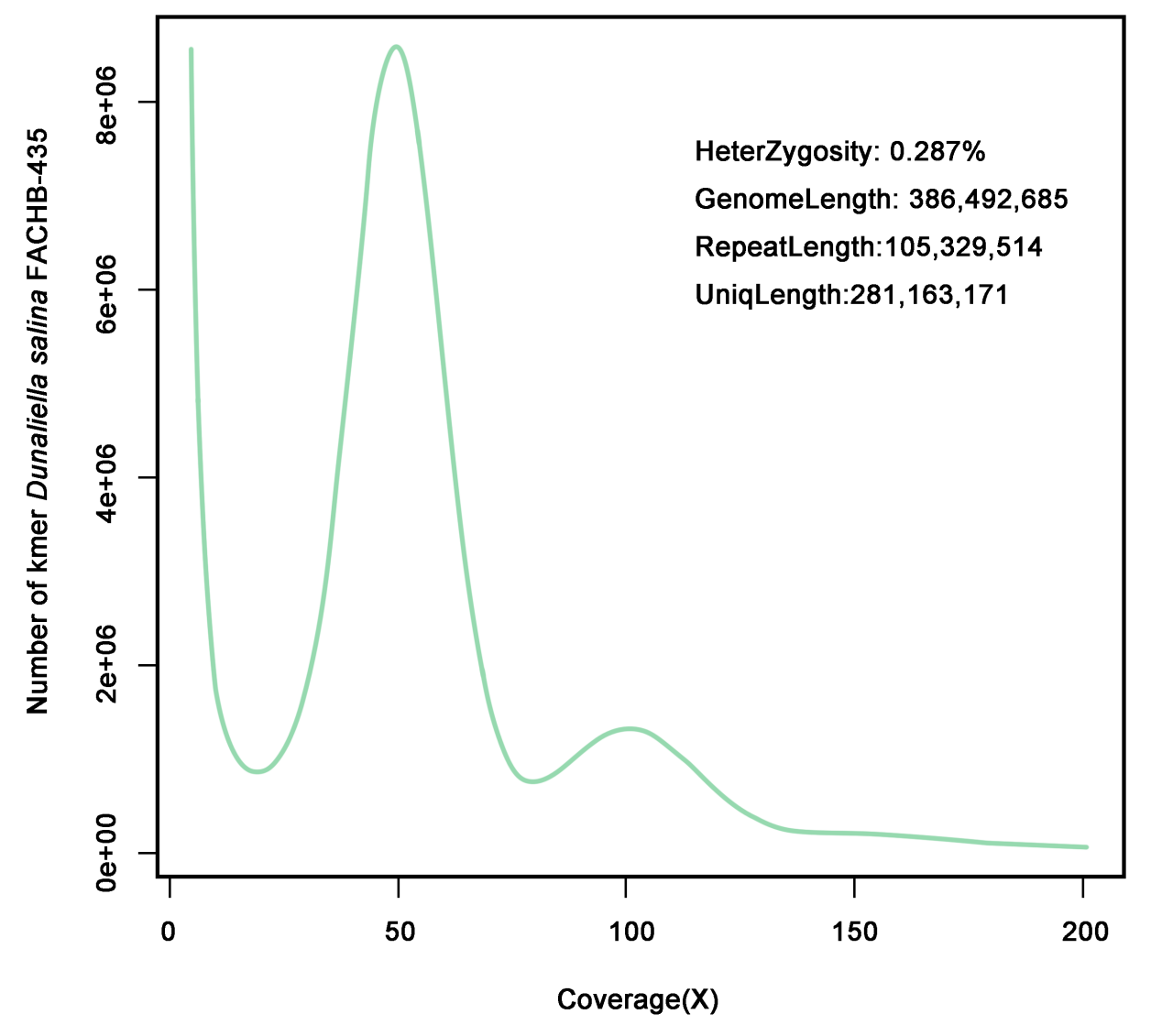


Fig.S1 Distribution of kmer frequence of *Dunaliella Salina* FACHB-435


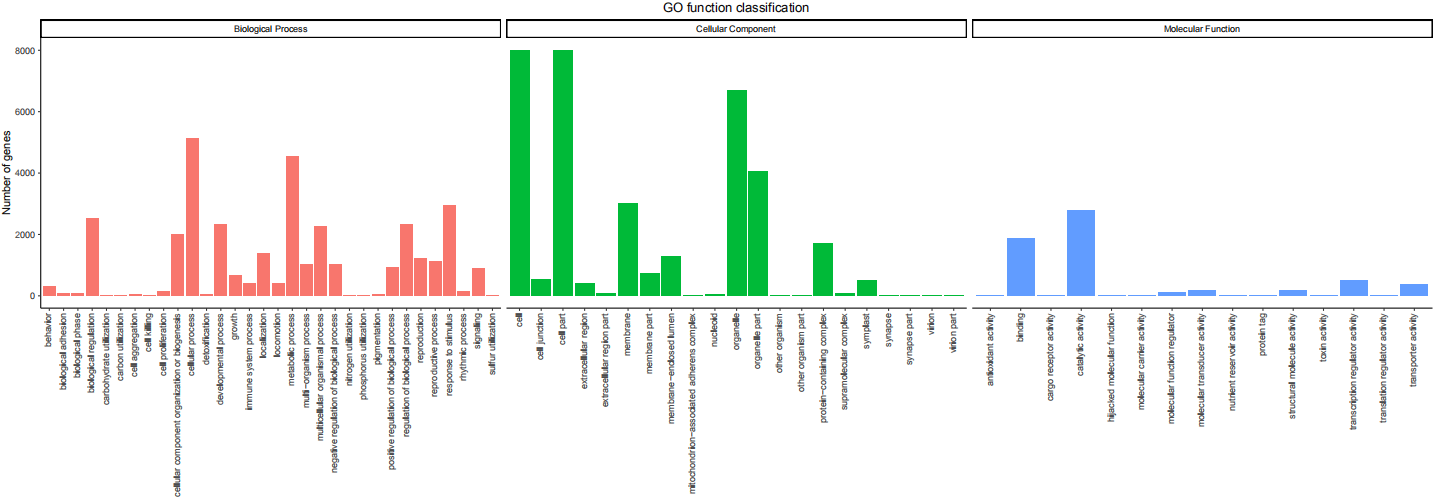


Fig. S2 Classification figure of GO


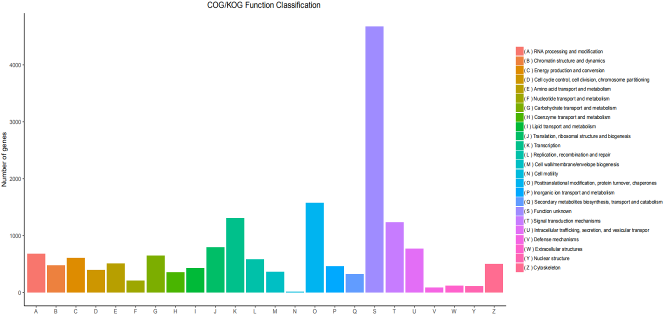


Fig. S3 Classification chart of KOG
